# Adaptive arbitration of aerial swarm interactions through a Gaussian kernel for coherent group motion

**DOI:** 10.3389/frobt.2022.1006786

**Published:** 2022-12-01

**Authors:** Tiziano Manoni, Dario Albani, Jiri Horyna, Pavel Petracek, Martin Saska, Eliseo Ferrante

**Affiliations:** ^1^ Autonomous Robotics Research Center Technology Innovation Institute, Abu Dhabi, United Arab Emirates; ^2^ Department of Computer Science Vrije Universiteit, Amsterdam, Netherlands; ^3^ Department of Cybernetics Czech Technical University, Prague, Czech

**Keywords:** unmanned aerial vehicle, flocking, field experiments and simulations, swarm robot control, swarm (methodology)

## Abstract

Swarm behaviors offer scalability and robustness to failure through a decentralized and distributed design. When designing coherent group motion as in swarm flocking, virtual potential functions are a widely used mechanism to ensure the aforementioned properties. However, arbitrating through different virtual potential sources in real-time has proven to be difficult. Such arbitration is often affected by fine tuning of the control parameters used to select among the different sources and by manually set cut-offs used to achieve a balance between stability and velocity. A reliance on parameter tuning makes these methods not ideal for field operations of aerial drones which are characterized by fast non-linear dynamics hindering the stability of potential functions designed for slower dynamics. A situation that is further exacerbated by parameters that are fine-tuned in the lab is often not appropriate to achieve satisfying performances on the field. In this work, we investigate the problem of dynamic tuning of local interactions in a swarm of aerial vehicles with the objective of tackling the stability–velocity trade-off. We let the focal agent autonomously and adaptively decide which source of local information to prioritize and at which degree—for example, which neighbor interaction or goal direction. The main novelty of the proposed method lies in a Gaussian kernel used to regulate the importance of each element in the swarm scheme. Each agent in the swarm relies on such a mechanism at every algorithmic iteration and uses it to tune the final output velocities. We show that the presented approach can achieve cohesive flocking while at the same time navigating through a set of way-points at speed. In addition, the proposed method allows to achieve other desired field properties such as automatic group splitting and joining over long distances. The aforementioned properties have been empirically proven by an extensive set of simulated and field experiments, in communication-full and communication-less scenarios. Moreover, the presented approach has been proven to be robust to failures, intermittent communication, and noisy perceptions.

## 1 Introduction

In this work, we focus on airborne systems such as quadrotors and unmanned aerial vehicles which we refer to as UAVs. In the last decade, the use of UAVs increased at a fast pace, and despite it being under actuated, such a platform design is very successful and represents a big part of the robotics market, nowadays attracting both research and industry. Amongst the many applications envisioned for it, drone shows are certainly the most impressive with 1,824 airborne units at the Tokyo Olympics and a record-breaking show of 3,281 set up by a luxury car retailer (Peters., 2021). However, impressive numbers, as reported in a recent review article by Coppola et al. (2020), are showcased in demonstrations that lack the decentralized and distributed properties of swarm robotics approaches. For example, centralization as opposed to decentralization: the controller runs on a single central computer rather than on-board of each drone; a centralized system not only poses the threat of a cardinal point of failure but also requires stable communication among the parties. Furthermore, the previously cited examples fully rely on external high-precision sensing such as visual tracking systems or real-time kinematic Global Positioning System (RTK-GPS), limiting their usability to structured environments and ruling out different field applications.

Among the many different building block behaviors that can be executed by an aerial swarm, in this work, we consider the basic problem of coherent group motion, still far from being considered solved in real-world scenarios. Throughout the work, we refer to this problem as flocking even though, as pointed out by Logan and Malikopoulos (2021), a problem arising from the literature is the use of such a term to summarize different modes of motion. Originally inspired by biological entities such as fish, birds, and even bacteria, flocking schemes have evolved and are applied to artificial entities—usually referred to with the generic term boid, shorthand for bird–android. We analyze the problem of designing self-organized flocking of unmanned aerial vehicles, UAVs, by putting the accent on robustness and scalability and at the same time, considering real-world applications. We are not the first to do this, and over the past years, the problem has been tackled from multiple perspectives, different models and formulations have been proposed, and impressive results have been achieved. Some of these works are presented and analyzed better in the following sections where we present an overview of the current state-of-the-art and compare it with the approach presented here.

The presented approach builds upon previous works (Ferrante et al., 2012; Ferrante et al., 2014; Amorim et al., 2021) and, in particular, on Albani et al. (2022), opting for a path different from other models that build over the so-called Vicsek model (Vásárhelyi et al., 2014; Vásárhelyi et al., 2018; Balázs et al., 2020) or others building on the Cucker–Smale formulation (Cucker and Smale, 2007). Among the many open challenges, we tackle the problem of dynamic tuning of the interactions between the focal agent and the different sources of local information—such as its neighbors and the environment—to enable faster and more stable flocking. This is carried out using a specifically designed Gaussian kernel on which every agent, in a distributed fashion, relies to automatically weight its neighbors and the target. This is the main conceptual contribution of this work, and it not only enables fast and stable flocking but also allows for more complex behaviors such as group splitting and joining. This work also possesses experimental contributions: first, we show how our approach reacts to a real-world and challenging scenario as the Abu Dhabi desert; next, we analyze long-range group splitting and joining behaviors along with an insight into performed experiments; and finally, insights about the use of the sensing technology based on ultra-violet cameras and its expected behaviors are also presented. Experiments and simulations were performed both in the presence and absence of communication, over long distance, and at an allowed top speed up to 8 m/s.

As anticipated, the following section focuses on the state-of-the-art and presents some related work. With a clear perspective of the current status of the research, we then introduce our proposed flocking controller in Section 3 and move to its performance analysis in Section 4. Here, both simulated and field results of a group of up to 10 robots are presented. In the same section, we also report real field experiments performed in the complete absence of communication and we present simulated results with a large group of 25 agents. The latter offers a comparison of the proposed approach against a state-of-the-art controller recently proposed. Section 5 closes the paper with conclusions and take-home messages.

## 2 State-of-the-art

The seminal work on flocking is the one by Reynolds (1987) from which a great part of the interest in flocking originated over the last decade. In this work, three heuristics are proposed: cohesion, agents in the swarm are attracted to the average position of respective neighbors; separation, robots are repulsed from neighboring robots; and alignment, each agent converges to the average velocity of its neighbors. These three heuristics set the foundation of future works (Crowther, 2004; Hauert et al., 2011; Ferrante et al., 2012; Amorim et al., 2021) and other sharing the concepts of attraction and repulsion in similar fashion (Vásárhelyi et al., 2014; Balázs et al., 2020). In the remaining part of this section, we first analyze the works that are more closely related to the contribution of this paper—primarily those focusing on flocking with UAVs. Then, we describe other literature reports that are less strongly related and that also encompass works on different robotic platforms such as UGVs. Finally, the section concludes by analyzing works in which relative localization systems, such as those used by our drones, are used.

Among the works that are more closely related to the seminal paper of Reynolds and the present paper, we find the one by Crowther (2004) and one by Hauert et al. (2011). Here, a fourth rule, migration, is applied to steer the swarm toward a wanted direction or migration point. In particular, in Hauert et al. (2011), each of the four rules generates a vector which is merged with the other, producing a single output from the flocking controller. During motion, each agent modifies its heading with an angular rate proportional to the difference between the current heading and the desired heading given by the controller output at each cycle. As shown both in simulation and with the use of fixed-wing unmanned aerial vehicles, over time, the robots converge toward a common direction and migrate to the migration point in a cohesive manner. From an experimental point of view, the state-of-the-art for quadrotor autonomous drones is set by Balázs et al. (2020) where an impressive number of 52 have been put to work. The peculiarity of this work is the introduction of a “will,” an agent-centric perception of the neighbors’ persistence related to a perceived level of leadership. The assumption is that this information is shared among the swarm, by means of a communication mechanism, to aid the alignment with the neighbors’ velocities and eventually rule out local fluctuations. There is a strong assumption that we try to relax in this work, by proposing a control algorithm shown to work in the complete absence of communication—see Section 4. In the study by Balázs et al. (2020), similar to its preceding work (Vásárhelyi et al., 2014; Vásárhelyi et al., 2018), the control method is composed of short-range potential repulsion, middle-range velocity alignment, and global position constraint for flocking and formation flights. The first two rules recall the original work by Reynolds on boids; the third, on the other hand, is a newly introduced mechanism used to maintain the flock within specific global boundaries to achieve a coherent motion which would not be attained otherwise. Works such as those by Ferrante et al. (2012)and Ferrante et al. (2014) seek to maintain specific positions during motion by “locking in-place” the members of the swarm by specifically designed controllers seeking the local minima of the surrounding forces. These works have been designed having ground non-holonomic robots in mind, and only in Amorim et al. (2021) do we see the original control scheme from Ferrante et al. applied to quadrotors. The work is based on virtual potentials that generate areas of minimum repulsion and attraction. Such local minima distribute in space-defining “locking” regions: a rhomboid shape in the case of four robots in a two-dimension space or a sphere-like arrangement for bigger swarms in three dimensions. A difference between the controller we describe in this work and that if of Amorim or Ferrante et al. is the absence of non-holonomic constraints. The absence is justified by a different objective: we aim to orchestrate between flocking and migration forces, and thus, we assume all agents to have a target, with no need for holonomic constraints in such a setup. Moreover, while in Amorim et al. (2021) and Ferrante et al. (2014) the robots are forced to turn in place and align toward the motion direction, we consider instantaneous motion on every axis and from that output a single velocity vector that defines the motion of the single. Moreover, even though we share repulsion and attraction rules, we dynamically relax the region of minimum force, relaxing the formation but improving the overall speed and reaction of the flock.

We conclude this section by analyzing other works carried out in the context of engineering self-organized flocking. In a survey, Logan and Malikopoulos (2021) proposed a partition in the engineering flocking literature. The authors proposed to divide the state-of-the-art into two big subsets, namely, line flocking and cluster flocking. The former aggregates all those works where the objective is to minimize the energy consumption of the entire swarm—similar to geese. The cluster flocking category, on the other hand, encompasses those works where the designer chases the optimization of a system-level cost function tailored to control the policy of each agent in the swarm. Other works rely on schemes similar to the aforementioned one, for instance, Ali et al. (2008), Kownacki and Daniel (2016), and Dmytruk et al. (2021). In 2018, Ali et al. (2008) presented a new robotic platform called Kobot, equipped with an infrared sensor for measuring the distance between robots and obstacles, and a sensor for perceiving the relative headings of neighboring robots. A behavior based on proximal control and heading alignment is then put in place on such a platform and shown to be capable of generating self-organized flocking in a swarm. The most recent of the aforementioned works is that of Dmytruk et al. (2021). The work presents a bio-inspired decentralized flocking algorithm working in environments with high obstacle density that only relies on local perceptions. The approach couples both obstacle avoidance, because of the use of Voronoi diagrams, and flocking in a single control scheme. On the other hand, Kownacki and Daniel (2016) built directly upon the work of Reynolds (1987). The peculiarity of this approach is the presence of informed leaders who act as guides for the whole swarm, steering the so-called followers in a coherent manner. This last work falls within a whole different sub-set of the flocking literature, studying leadership in flocking. This is also true for approaches such as those by Hung and Givigi (2016) and Yan et al. (2021) that apply reinforcement learning to the leader–follower flocking problem. Hung and Givigi (2016) uses Q-learning in a Markov decision process where the agents are modeled as small fixed-wing UAVs. Yan et al. (2021) used deep reinforcement learning instead to teach collision-free policies for a scalable fixed-wing UAV swarm. Both works share a similar reward function consisting of a flocking reward that encourages the followers to maintain the desired distance from the leaders and a collision penalty. Overall, the leader vs. leader-less problem has been studied both in biological settings in birds (Bajec and Heppner, 2009) and in artificial settings on boids (Jia and Vicsek, 2019). The conditions under which leader-driven cluster flocking is optimal are an open question and we leave it out for the remainder of this work as it is not related to the approach we present.

We conclude the state-of-the-art section by analyzing a few works that rely on relative location in a similar fashion as we propose here in this work. Coppola et al. (2018) used blue-tooth antennas to communicate velocities, altitude, and orientation and estimate the range using the signal strength. The authors then used this information to build a navigation scheme for quadrotors and prove how it is possible to achieve a collision-free flight on a small swarm of three robots. A different approach is the ultra-violet light detection and ranging system, UVDAR (Walter et al., 2018; Walter et al., 2019). As shown by Petracek et al. (2020) and in this work, this system can be used for estimating the relative position of surrounding robots. The system consists of UV LED markers placed at the quadrotors’ edges and UV-sensitive cameras covering as much field of view as possible. Classic computer vision techniques are then used to extract the position of the other agents from the markers perceived by the ultra-violet cameras. More details about the system are given in the following sections as we introduce the hardware setup for the real-robot experiments.

## 3 Flocking controller

Overall, the proposed control architecture is an evolution of the one presented in Albani et al. (2022) but also shares similarities with Hauert et al. (2011). The main difference between this approach and the aforementioned one and others (Vásárhelyi et al., 2014; Vásárhelyi et al., 2018; Balázs et al., 2020) is that we do not seek velocity alignment. The absence is compensated by the use of a Gaussian mixture specifically designed for the scope. Furthermore, we do not rely on the friction component used in some of the aforementioned works to over-damp the velocities, and no global constraints are in place other than the migration force steering the swarm toward the target.

Consider a robot *n*_*i*_ in the set of robots *N* composing the swarm, with *i* ∈ *N* used to identify a single unit. Let the robots’ position be denoted as $\vec{x_{i}}\in R^{d}$ with *d* representing the dimension of the state space on which the robots are moving—that is, *d* = 3 for aerial robots and *d* = 2 for ground robots. Our flocking controller ensures coherent motion by a set of control inputs $\vec{u_{i}}\in R^{d}$ so that ${\vec{x_{i}}}^{t+1}={\vec{x_{i}}}^{t}+B^{t}{\vec{u_{i}}}^{t}$ , with *B* ^*t*^ being the control input model, identical for all robots, and therefore omitted in the following. With these assumptions, for a generic robot *i*, the proposed flocking controller writes

$$\vec{u_{i}}=w_{i}\underset{j\in \hat{N}}{\sum }\vec{n_{j}}+\left(1-w_{i}\right)\vec{t_{i}}+\vec{g_{i}}.$$ (1)

Each term in Eq. 1 identifies one of the three different components of the proposed approach which are analyzed in detail in sub-Section 3.2: $\vec{n_{i}}$ is defined as the proximal control vector and is used for the neighbors’ proximal control, governing the distances between the robots; $\vec{t_{i}}$ is the target proximal control vector for the *i*-th robots and is used to steer the swarm toward the target; and $\vec{g_{i}}$ is the ground proximal control vector which, as the name suggests, keeps the swarm at a wanted altitude from the ground. The sum range $\tilde{N}\subseteq N$ is defined as the subset of agents currently perceived by the focal agent. The number of perceived neighbors is not predefined, is not capped, and only depends on the technology used for the task with no assumption on it—for example, communication devices and electro-optical sensors provide different performances. Lastly, the variable *w*_*i*_ is called the importance weight and represents the main novelty introduced in this paper.

### 3.1 Importance weight

The role of *w*_*i*_ is to tune the contribution of the different components in Eq. 1 in a way that fast and group-coherent motion toward the target is achieved. This is a common problem when designing flocking algorithms (Balázs et al., 2020; Albani et al., 2022) where fast motion toward the target is desired but at the same time, the swarm should take care to avoid collisions. This trade-off is crucial for many real-world applications and what we propose here is an alternate solution to the problem. In the proposed formulation, the importance weight is computed by means of a mixture of Gaussian functions as the one shown in Figure 2 and is expressed as follows:

$$W\left(\rho \right)=\left\{\begin{array}{c} 1-N\left(\rho ,\mu_{1},\sigma_{1}\right)\;\;if\;\rho \leq d\;\\ \frac{1}{2}\left(1-N\left(\rho ,\mu_{2},\sigma_{2}\right)\right)\;\;if\;d<\rho <2d\;\\ \frac{1}{2}\left(N\left(\rho ,\mu_{3},\sigma_{3}\right)\right)\;\;if\;\rho \geq 2d\; \end{array}\right.$$ (2)

**FIGURE 1:**
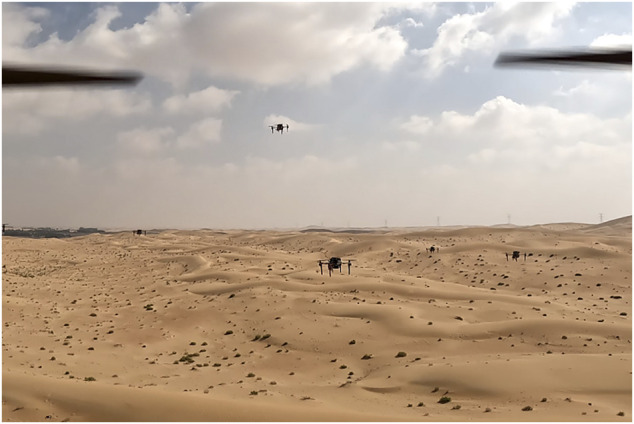
Aerial point of view of a swarm unit in the test location in the Abu Dhabi desert during the experiments.

**FIGURE 2:**
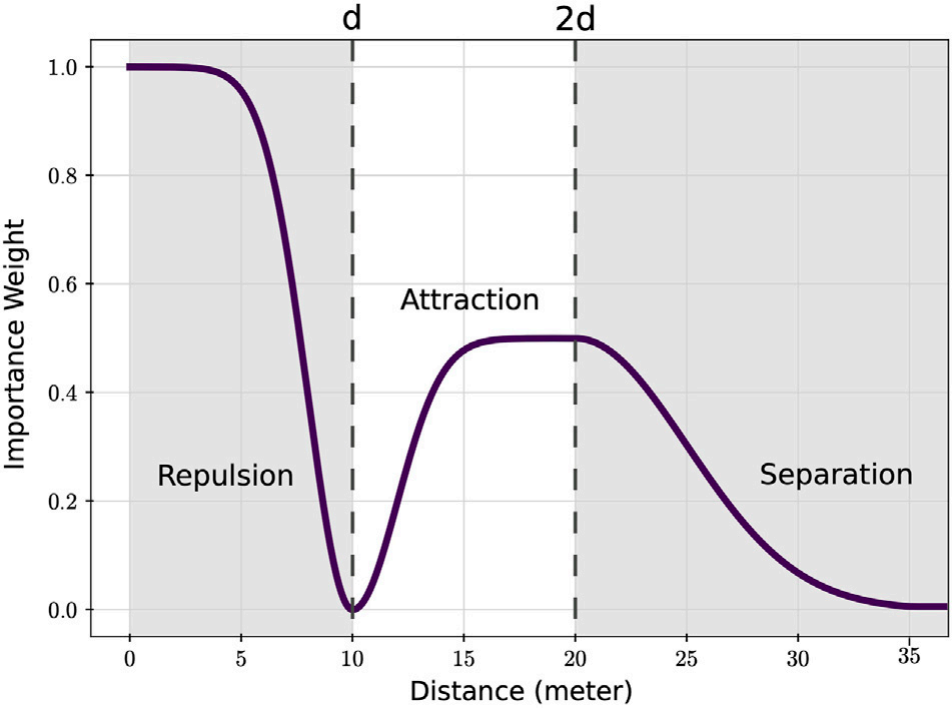
Gaussian mixture in Eq. 2 used to compute the importance weight. The three highlighted regions in the plot identify different roles of the importance weight function. Parameter *d* is the desired distance of the focal robot from the interest point.

Here, *ρ* is the function sampling point, and *σ* and *μ* represent the variance and the mean, respectively, of the respective Gaussian $N=e^{-{\left(\rho -\mu \right)}^{2}/\left(2\sigma^{2}\right)}$ . The parameter *d* represents the wanted distance from the sampling point and is analyzed in more detail as follows. The aforementioned set of equation builds up the Gaussian mixture *W*(*ρ*) used to compute the importance weight *w*_*i*_ in Eq. 1 as follows:

$$w_{i}=\underset{j\in \hat{N}}{\sum }W\left(\rho_{j}\right),$$ (3)

where the sampling point *ρ*_*j*_ is the distance of the focal robot from its j-th neighbor drone. Thus, for every algorithmic cycle, the focal robot proceeds with the computation of the virtual potentials and the assignment of the weights to the neighbors in a non-linear relation to their position. The idea behind this concept can be easily understood from the graph in Figure 2. In the plot, *d* = 10 is again the wanted distance from the sampling point—that is, in the flocking case, from a neighbor. The three regions marked as *repulsion*, *attraction*, and *separation* identify the three different Gaussian functions that build up the mixture and whose role is defined by the respective label. The first region from the left is designed to provide strong importance to agents that are closer than the wanted distance reaching a null contribution at *d* and a peak at 0 distance—that is, imminent collision. The central area, denoted as attraction and defined from *d* to 2*d*, again has null contribution at the desired distance and increases up to a value of 0.5 importance as the distance increases, ending in a plateau two times the desired distance. The rationale here is to enforce an intermediate consideration of the agents within this range providing a slack formation that, however, still secures coherent motion. The last stretch of the function is named the separation phase, as shown in Figure 2, and it is defined for a distance greater than two times the desired one. In this phase, a dynamic and smooth cut-off takes place. This is an important region that allows the smooth separation and re-joining of sub-groups but, at the same time, is also crucial in a large swarm to reduce oscillation and avoid the unwanted disengagement of the focal robot from the group. A more detailed analysis aided by simulated swarms of the aforementioned concepts is provided in Section 4.5.

### 3.2 Proximal controls

Henceforth, to favor clarity in the description of the algorithm, we assume the robot frame of reference to be attached to its flight control unit and described by the right-hand rule. The horizontal plane formed by the x-axis and y-axis is parallel to the ground and not tilted with respect to the latter. The z-axis is perpendicular to the horizontal plane and coherent with altitude readings: positive z-values as the drone ascends and negative as it descends. Robot positions are not expressed in Cartesian coordinates but rather by using spherical coordinates: a position in space represented by a triplet of scalars (*ρ*, *θ*, *ψ*) where *ρ* identifies the norm of the vector starting from the origin and pointing toward the desired position—also called radial distance—and is the same as that used in Eq. 3; *θ* represents the counter-clockwise rotation on the horizontal plane; and *ψ* is the rotation from the initial meridian plane. The aforementioned conventions are dictated by the need of abstracting sensory readings for them to match among different hardware. As an example, in this paper, we test both with UV camera readings and GPS readings but this is completely transparent to the proposed approach.

Next, we introduce the first of the three proximal control components defining the rules of attraction and repulsion for each robot in the swarm. The first, acting between the focal robot *i* and its neighbors, is called neighbors’ proximal control and can be described as a pair potential. The role of this component is to balance attraction and repulsion forces, acting as a virtual spring and keeping the required separation among the units. The proximal control is expressed as follows:

$$\vec{n_{i}}=\underset{j\in \tilde{N}}{\sum }f_{n}\left(\rho_{j},\theta_{j},\psi_{j}\right)$$ (4)

The function *f*_*n*_(⋅)—with ⋅ shorthand for the spherical coordinates triplet (*ρ*_*j*_, *θ*_*j*_, *ψ*_*j*_)—uses the Lennard–Jones virtual potential (Ferrante et al., 2014; Amorim et al., 2021) to compute single-axis components with the same parameters previously investigated in Albani et al. (2022). The Lennard–Jones virtual potential is written as follows:

$$L_{n}\left(\rho \right)=-\frac{4\alpha \epsilon }{\rho }\left[2{\left(\frac{\sigma }{\rho }\right)}^{2\alpha }-{\left(\frac{\sigma }{\rho }\right)}^{\alpha }\right]$$ (5)

The parameters *α* and *ϵ* are set to 1.2 and 3, respectively, following previous works of Ferrante et al. (2012) and Amorim et al. (2021). The term *d* is again the desired distance that is used to center the potential, while the variable *ρ* represents the sampling point. This allows us to use a single virtual potential both for attraction and repulsion forces and to generate single Euclidean axis velocities as follows:

$$f_{n}\left(\cdot \right)=\left\{\begin{array}{c} f_{n}^{x}=L_{n}\left(\rho \right)*\cos\left(\theta \right)*\sin\left(\psi \right),\;\\ f_{n}^{y}=L_{n}\left(\rho \right)*\sin\left(\theta \right)*\sin\left(\psi \right),\;\\ f_{n}^{z}=L_{n}\left(\rho \right)*\cos\left(\psi \right).\; \end{array}\right.$$ (6)

Superscripts *x*, *y*, *z* indicate the axis of application of the computed force, L indicates the Lennard–Jones virtual potential, and finally *ρ* and *θ* are the same angles used in Eq. 4 with the *j* subscript dropped for ease of interpretation. With respect to Ferrante et al. (2014), Amorim et al. (2021), and Albani et al. (2022), no manual cut-off over the distance is in place. Indeed, in the previous approaches, a parameter was used to arbitrarily decide when to cut the influence of the pair potential between two units, whereas in this study, we relax the need of fine tuning. This is because of the presence of the importance weight. The latter, introduced in Sub-section 3.1, automatically tunes the interactions between agents in the same group, removing the need of a cut-off. The role of Eq. 6 is to project the force magnitude as output along the three Euclidean axes of motion, generating single-axis velocities. The function L is centered at a specific desired distance *d*—one of the few design parameters present in this work—that is the same used in Eq. 2, representing the wanted desired gap between two robots. In Eq. 1, the neighbors’ proximal is balanced due to the use of the importance weight whose role is to find a dynamic trade-off between intra-swarm motion needed for formation control, and the swarm flows toward a generic target.

The migration of the swarm is controlled by the target proximal and is used to steer the unit—and the overall group—toward the wanted location.

$$\vec{t}=f_{t}\left(\rho ,\theta ,\psi \right).$$ (7)

Again, the input triplet expresses the target position relative to the focal robot and the function *f*_*t*_(⋅) determines the target attraction and repulsion components. The desired distance *d*_*t*_ from the target in the case of way-point navigation is set to zero, while in other scenarios, it can be non-null to avoid colliding with the target.

Finally, the last term in the flocking controller equation is in charge of arbitrating the distance of the swarm from the ground. The ground proximal control considers the terrain as a single point of contact and is defined similar to its antagonist neighbors and target proximal control:

$$\vec{g}=f_{g}\left(\rho ,\theta ,\psi \right).$$ (8)

Again, the function *f*_*g*_(⋅) produces output velocities computed using the Lennard–Jones virtual potential set to respect a desired altitude.

## 4 Swarm experiments

We start investigating the performances of the proposed controller by illustrating real-world experiments and their simulated counterpart, and then perform a more in-depth simulation-based analysis. Simulations were performed using the Flightmare simulator (Song et al., 2021). Field tests have been performed in the Abu Dhabi desert over two different experimental campaigns, in summer and fall. Due to the harsh conditions presented by these settings, hardware failures have been looming throughout the campaign: drone frames bending due to the hot weather and sun, the GPS receiver drastically diminishing its accuracy at sunset, and altitude estimator failures causing the drones to crash. Nonetheless, as thoroughly analyzed in the following sections, the proposed approach is proven to be dynamic, repeatable, and robust to unit malfunctions.

### 4.1 Metrics

For a formal evaluation of the experimental results, both simulated and real, we propose different metrics. First, we evaluate the cluster approach velocity or CAP, which expresses the ratio of the average velocity of the cluster *C* while approaching a given way-point. The CAP is expressed in relation to the maximum velocity input by the operator *V*_max_, which results in values ranging between 1—the average velocity for the cluster is equal to *V*_max_—and 0—no motion. It is expressed as follows:

$$CAP=\frac{\sum_{i\in C}\vec{V_{i}}\sin\theta_{{wp}_{i}}}{{\vec{V}}_{\max}}$$ (9)

The term *V*_*i*_ is the i-th agent velocity re-scaled with respect to the angle pointing toward the way-point $\theta_{w_{i}}$ as seen from the agent *i*. Moreover, with the term cluster, indicated as *C*, we indicate a group of connected robots. Two robots are considered connected under two conditions: 1) both robots cannot perceive each other—either because they are outside the visual or communication range or 2) the value of the importance weight depicted as the Gaussian mixture in Figure 2 is below the arbitrary value of 0.05—that is, the force generated from any of the two robots affecting the other is negligible.

As a second metric, we define the *COR*—correlated unit flocking velocity—that we use to analyze the relation between the single robot velocity $\vec{V_{i}}$ and the cluster average velocity $\vec{V_{\bar{c}}}$ with $\bar{C}$ , identifying the reduced cluster *C* without considering the focal agent *i*. In contrast to the cluster approach velocity that refers to the performance of the whole cluster, the correlated unit flocking velocity is computed for each agent. It is expressed as follows:

$$COR=\frac{\vec{V_{i}}\cos\theta_{\bar{C}}}{{\vec{V}}_{\bar{C}}}$$ (10)

The term $\theta_{\bar{C}}$ represents the angle between the cluster and the robot motion vectors. We also note that the cluster motion vector might not be aligned with the way-point when the COR is high—that is, the group CAP can be lower than 1 when the COR is very close to 1. Nonetheless, this metric is useful to separate the cluster motion, expressed with the CAP and originated from migration forces, from possible oscillations of the single robot induced by the flocking controller. To this end, we expect a value of 1 for the COR to indicate an optimal velocity alignment between a single unit and the swarm, while more commonly, in real experiments, values are expected to oscillate around it.

As the third and fourth metrics, we report both the minimum and the average distance among the agents in the cluster. These are important to spot near-misses and collisions and to understand the effect of the importance weight in the proposed flocking controller.

### 4.2 Hardware setup

**FIGURE 3:**
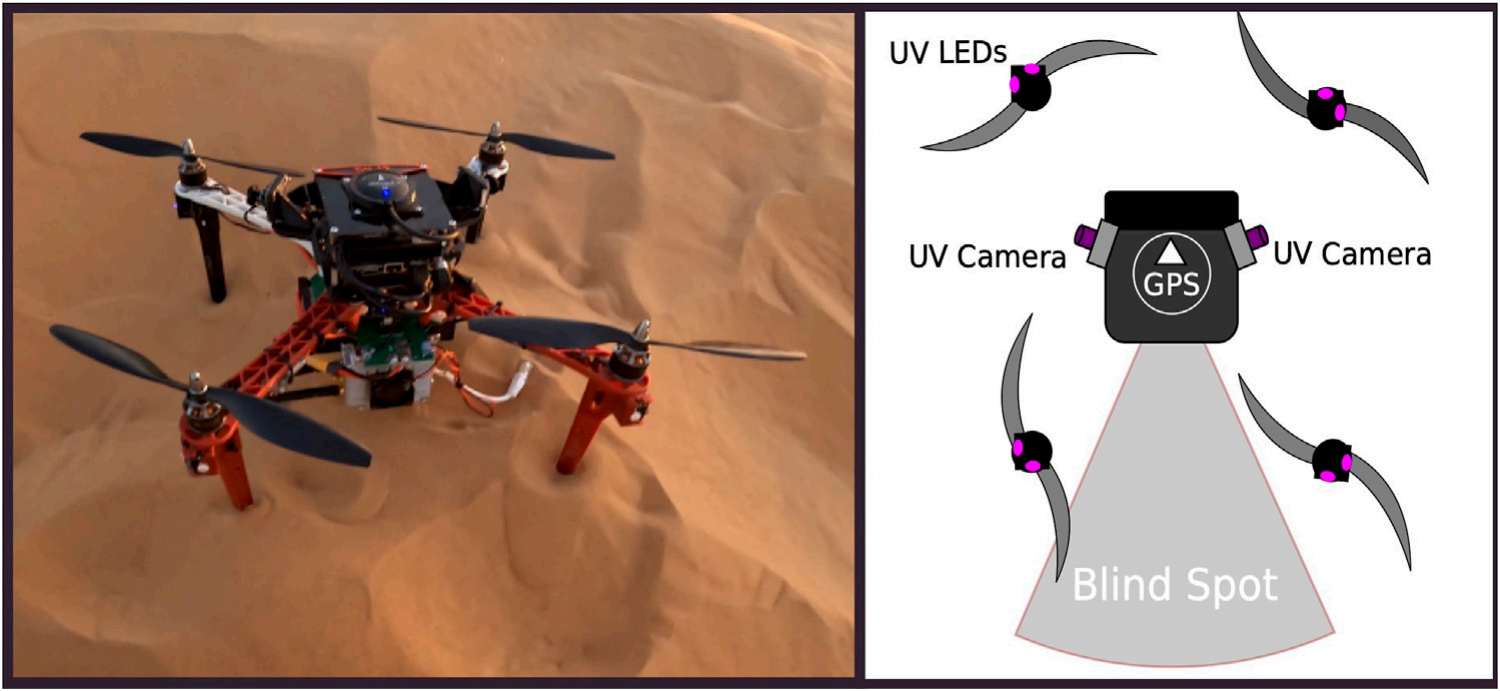
(Left): a picture of the real hardware used during the experiments; (right) an abstract representation of the UVDAR setup used during the communication-less experiments. In the left figure, the following elements can be recognized: Mobilicom MCU-30 device—gray box on the bottom of the drone; UV LEDs—small white LEDs located at the edge of each arm; UV-sensitive camera—on the right and the left of the drones peeking out the main body.

The platform of choice for the field experiments is the MRS450, developed by the company Fly4Future and running the MRS UAV-system researched by the multi-robot systems group at the Czech Technical University in Prague (Baca et al., 2021). It is shown in Figure 3-left. The custom platform consists of a DJI f450 frame with four rotors, a Pixhawk 4 autopilot, and an Intel NUC with an i7 Intel processor as an on-board computer. From a sensory point of view, the drone is equipped with a Garmin LiDAR Lite-V3 rangefinder for heights above ground estimation, a standard Global Positioning System module for self-localization. Communication in real-world scenarios uses an ad-hoc peer-to-peer network among the agents and from the swarm to the ground control station (GCS) for telemetry—always present for scientific purposes in communication-less experiments as well. The physical and network layers are provided by the 2.4 GHz radio-based devices *Mobilicom MCU-30 Lite* mounted on-board the UAVs and by the *MCU-200* plugged for monitoring purposes. We used omni-directional low-power antennas on-board all UAVs, while high-power antennas were used on the GCS to ensure continuous communication with the robots for safety reasons. Under this condition, the drones rely on a one-hop mesh network built on a fully connected graph. No centralization or off-load of computations from the swarm to the GCS of any sort was put in place during the experiments. To analyze the performances of the proposed approach in the absence of communication, we installed on the UAVs the ultra-violet direction and ranging (UVDAR) system (Walter et al., 2019). The UVDAR is a relative mutual localization system that enables estimation and tracking of relative positions of the surrounding UAVs. The hardware components that build up the system are a set of ultra-violet LEDs mounted below the propellers, and two ultra-violet-sensitive cameras were placed as shown in the schematized representation of the drone in Figure 3-right. The setup relies on two cameras that together provide a 320° horizontal and 110° vertical field of view, leaving a 40° horizontal blind spot on the back of the drone that was found sufficiently good for the swarming experiments. The concept behind the UVDAR is to operate in the UV spectrum due to primary natural light sources—such as the sun and its reflections—emitting less radiation in the chosen spectrum than in the visible light. This generates a black-and-white image as the output that is mostly black and is easy to process with computer vision techniques. As with all the other software used for the experiments, the software stack for the UVDAR runs completely on-board each unit in the swarm and uses computer vision algorithms to estimate the relative position of the blinking UV LEDs of the neighbors.

### 4.3 Connected swarm

We start with the analysis of the real field experiments in a communication-full scenario by exploiting the hardware setup presented in the previous section. In all the communication-full experiments, both real and simulated, each element of the swarm only shares its own global position *via* broadcast. When a neighboring robot within the range receives such information, it translates it in its own local reference frame and feeds it to the proposed controller. Indeed, the drone’s initial, intermediate, and final positions are always expressed in respective relative coordinates for each unit, and navigation way-points are provided to each drone separately and in a distributed fashion. For the real experiments, we opted for a static network scenario. No physical obstacles other than the aerial units were present and with the bandwidth distributed uniformly among up to 12 communication units. In such conditions, the observed communication persisted below 100 ms for all UAV–UAV and UAV–GCS connections. In this scenario, the maximum communication distance of the setup was empirically evaluated to 650 m—that is, the GCS fully stopped receiving telemetry information from the swarm after approximately 650 m.

**FIGURE 4:**
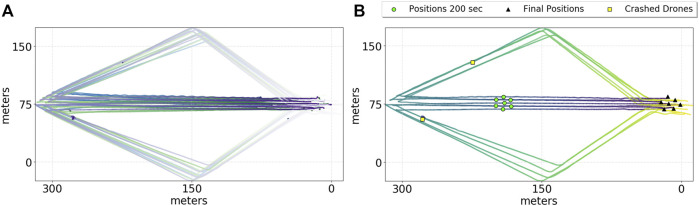
Trajectories of 9 UAVs performing a diamond-shaped trajectory during real experiments. The color of the trajectory becomes lighter with the flow of time; a lighter color represents the initial phase, while a darker color denotes the final phase of the experiments. The top plot **(A)** shows a superposition of the trajectories performed by each UAV over three different experiments. The bottom plot **(B)** analyzes one single particular experiment where two different crashes on two different units were experienced and highlighted here as yellow squares.

Trajectories from the performed experiments are shown in the plots in Figure 4 from which the repeatability of the proposed solution can be immediately appreciated. The top plot presents a top-down view of a set of three experiments with the swarm performing a diamond-shaped trajectory while splitting and merging along it. Lines in the plot define the trajectories of different robots over each run. All the robots share the same settings and the flocking controller used is the same as in Eq. 1. The variances of the Gaussian functions used for the importance weights in Eq. 2 are *σ*_1_ = 2, *σ*_2_ = 4, and *σ*_3_ = 6, respectively. Drone nominal velocities are set to a maximum of 4 m/s for real experiments and 6 m/s—larger velocities are investigated in Section 4.5. The only exceptions are the provided way-points: to achieve the separation and merging of the swarm, we provide two different sets of way-points to the two different sub-groups.

The bottom plot in Figure 4 shows a detailed view of a single run and focuses on the robustness of the approach by analyzing the case where 2 out of the 10 robots deployed underwent a critical system failure—a faulty barometer in one case and a faulty motor in the other—and crashed from an altitude of 25 m—yellow dots in Figure 4B. It shows how the trajectories of the swarm after the crashes are slightly, if not at all, affected by the latter, showing the robustness of the approach tested on the field. In fact, the rest of the swarm continued the mission even though two elements of the swarm were missing, by adapting the formation based on the number of drones left—green dots in Figure 4B.

**FIGURE 5:**
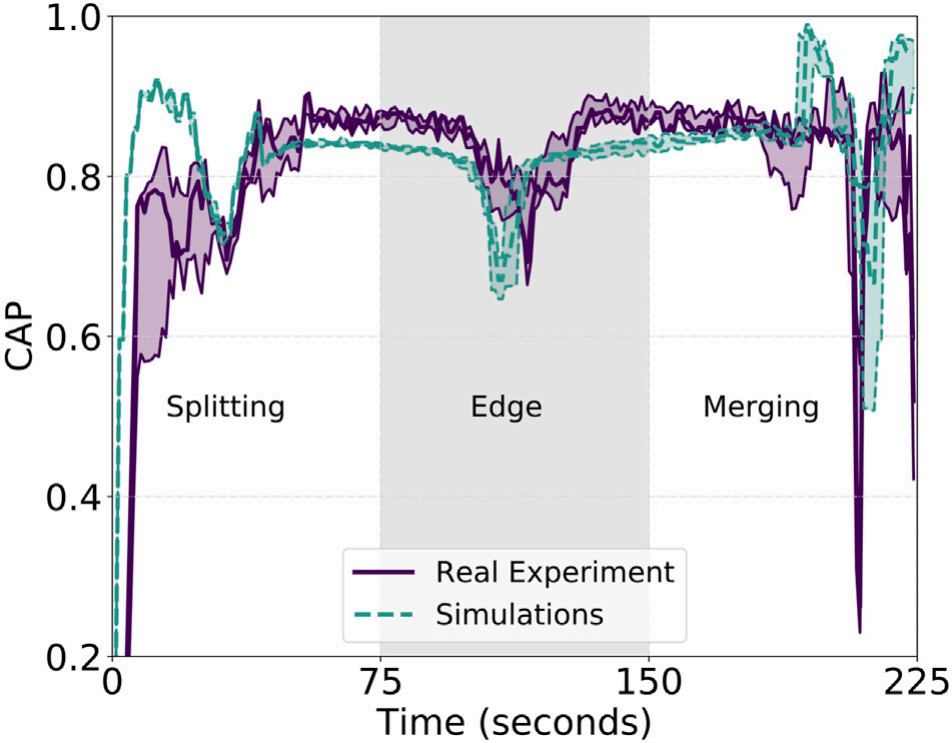
Reported CAP metric for the simulated and real experiments with communication. Higher y-values show better CAP performance. The metric is not computed for the whole experiments but only from the start until the group reaches the merging phase at roughly 400 m from the starting position. The last straight returning phase after the merging does not provide additional information and is left out for the sake of clarity.

**FIGURE 6:**
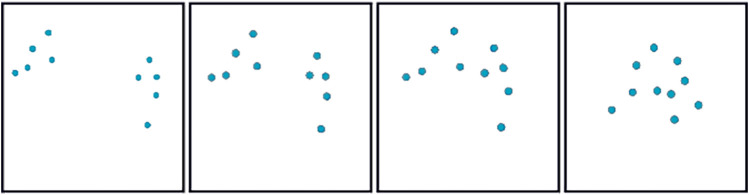
Four consecutive snapshots from a video of the experiments performed with communication in the loop. Each snapshot captures an aerial top-down view of the swarm during the “merging” phase. The images have been changed in color for readability purposes and provide an illustrative example of two sub-swarms merging into one at the end of the diamond-shaped trajectory designed for the experiments. For the original video, see the supplementary material.

We then compute the previously introduced metrics with respect to this particular set of experiments. The purple solid line in Figure 5 shows the computed average of the CAP over the three real-world experiments. From this, one can appreciate the balancing role of the Gaussian kernel tuning the importance weight coming from Eq. 1. Due to this, each robot in the swarm actively tunes its own speed based on the position of the neighbors, trying to generate an emergent behavior that keeps a consistent approach velocity toward the target. During the first phase, in Figure 5, the swarm accelerates and each unit reaches up to 90% of the maximum velocity *V*_max_. After a short trait, the “splitting” is initiated generating two different sub-groups heading toward different way-points. The importance weight kicks in as a reaction to the formation being broken and reduces the overall velocity of the swarm down to 70% of *V*_max_. The latter is an expected and required behavior to enforce a safe splitting maneuver, and it is easily explainable by the visual analysis of Figure 2 and its mathematical representation. In other words, we sacrifice the overall group speed for safety. This is carried out in complete autonomy, with no operator intervention, and takes place only when a particular maneuver—as merging and splitting—is performed. During the creation of the two sub-groups, all the robots are still within the sensing range and within the consideration phase—that is, in the attraction phase where *d* < *ρ* < 2*d*. Here, the overall value of *w* increases, reducing the migration forces in favor of the neighbors’ consideration. However, as soon as the two groups begin to form, the importance weight decreases again, allowing coherent and fast group motion. The two sub-swarms start accelerating again and reach a consistent CAP toward the next way-points, one of the two “edges” of the diamond. This phase is characterized by a sudden turn of almost 90° that causes an imbalance in the formation. As expected—but in this case, not desired—the importance weight starts increasing again, and the overall CAP decreases and then increases right after the formation is re-established. The swarms speed up again until the “merging” phase is reached. Figure 6 shows a series of consecutive snapshots taken from the video provided as the supplementary material, and serves as a visual illustration of the sub-swarms re-joining during one of the real experiments. The two groups are forced to re-join by their respective migration forces and approach each other from a frontal direction. We observe a non-negligible decrease in the speed of each group, a deceleration generated by the regained consideration of the other half of the swarm. In opposition to the “edge” phase where the action of the importance weight needs mitigation to allow for faster change of directions, during the “merging” phase, the reduction in the velocity is a wanted behavior ensuring collision-free trajectories.

**FIGURE 7:**
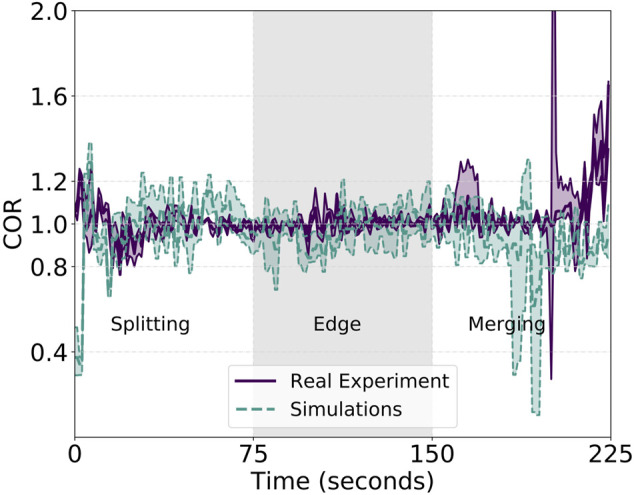
Reported COR metric for the simulated and real experiments with communication. Close to 1 y-values show better COR performance. The metric is not computed for the whole experiments but only from the start until the group reaches the merging phase at roughly 400 m from the starting position. The last straight returning phase after the merging does not provide additional information and is left out for the sake of clarity.

Additionally, Figure 7 shows the second of the proposed metrics: the correlated unit flocking velocity or simply COR. Being an indication of the velocity alignment between the unit and the swarm, a stable value as close to 1 as possible is desired. However, similar to the cluster approach velocity, the COR is affected by the three different phases of the experiment. On average, we measure an absolute value for the error of the COR—displacement from the optimal value of 1—of about 0.085, a value that is quite low and, therefore, confirms the robustness of the approach. We, however, observe peaks in the “splitting,” “edge,” and “merging” phases. In between these particular cases, characterized by either a group change of motion or group velocities colliding—as for the case of the merge, two low-variance zones are present. Associated with the two straight paths connecting the start with the edge and the edge with the end of the diamond, these two low-variance zones are useful to analyze the velocity alignment of the swarm during stable group flight. Overall, the analyzed unit velocity is aligned with the rest of the swarm among all three different runs but oscillations are still present. Indeed, the purple solid line in Figure 7, representing the real experiments, oscillates around the optimal value of 1. It is an indication of a slightly sub-optimal balancing effect of the controller and, in particular, of the importance weight. Acting as some sort of gradient descent looking for the minima in the Gaussian kernel, the importance weight fails in perfectly achieving an optimal and stable formation. This is due to several factors, analyzed better in the conclusion section of this work, such as noise, delayed communication and actuation, and a low-rate control step. Nonetheless, as presented in the following, the proposed controller does an extremely good job in balancing the swarm velocities even in non-trivial situations such as splitting, merging, turning, and robot failures. The latter is particularly visible in the COR plot at approximately 160 s. A peak in the variance of the COR is clearly visible indicating a strong velocity misalignment—due to one robot crash—that is immediately recovered afterward.

To strengthen the aforementioned statements, we back up the analysis of the proposed approach not only with field experiments but also with an extensive evaluation in simulation. For the simulated experiments, we increase the nominal speed by half and push the swarm to perform the same diamond trajectory with a *V*_max_ of 6 m/s. CAP and COR metrics for this set of experiments are shown in Figures 5 and 7, respectively, and identified by a dashed green line. While the cluster approach velocity is characterized by a lower variance than its real-experiment counterpart, the correlated unit flocking velocity presents higher variance. Indeed, the error for the latter is 0.17, double the real experiment value. This increase in error is expected because it is induced by higher admitted velocities the effect of which is mitigated by the importance weight. This is shown in Table 1 for both simulated and real experiments. The table reports the average of the distances and their standard deviation among the units during the tests. The results indicate that in the presence of communication, the average displacement from the desired inter-agent distance of 10 m is lower than the observed GPS noise—measured to be 0.8 m. Moreover, the simulated experiments also confirm the other properties highlighted in the field experiments. Overall, the simulations are very representative of the field experiments, which motivates the use of the simulator to perform the large swarm investigation presented in Section 4.5. The proposed controller does a good job in the automatic tuning of the migration and swarm forces, presents good repeatability, allows for non-trivial behavior, and at the same time, produces consistent and aligned velocity outputs for the single units in a distributed and decentralized manner.

**TABLE 1:** Reported average distance between drones in the swarm and its standard deviation during simulated and real experiments with communication.

	Average	Standard deviation
Communication (sim)	10.48	2.03
Communication (real)	9.6	2.82
UVDAR	9.8	2.82

### 4.4 Communication-less swarm

**FIGURE 8:**
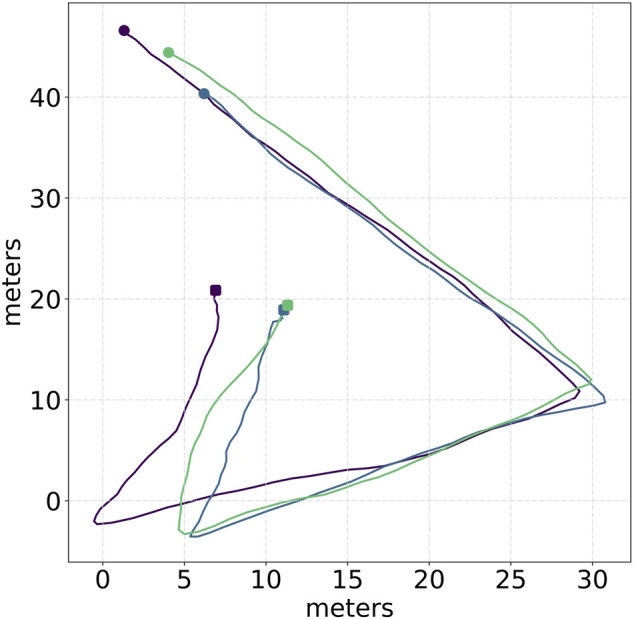
Triangle-shaped trajectory performed in real experiments with 7 UAVs equipped with UVDAR sensing technology. The picture shows the trajectory of the center of mass of the swarm over three experiments; different colors are associated with each experiment. A circle represents the starting point, while a triangle represents the endpoint of the trajectory.

We now aim at testing the adaptability of our algorithm to different conditions and its sensor abstraction capacity, that is, how easily different sensors and readings can be integrated with the approach. To this aim, we perform further real-world experiments in the complete absence of drone-to-drone communication with increased sensor noise and perceptions gaps. To this end, we use the UVDAR sensor described in Section 4.2 to have drones estimate neighbors’ relative positions directly on-board. As discussed in Section 4.2, the UVDAR technology produces different sets of reading but that can, as for the GPS, be easily adapted to work with the proposed approach. We also note that the precision of the UVDAR technology is lower than that of the Global Positioning System and decreases super-linearly with the error from the optimal perception distance. For these new sets of experiments, we propose a different and shorter trajectory with no splitting and merging behaviors. The triangle-shaped trajectory used for these experiments is shown in Figure 8 where we report three different runs performed with 7 robots.

Lastly, the hardware setup used for the experiments—as shown in Figure 3—presents a gap in the perception of 40°, not allowing the drone to perceive any of its neighbors on the back. This not only has the effect of hiding specific neighbors but also creates fluctuations to the potential functions when one or more units are at the edge of the perception, entering and exiting the sensing field and, thus, adding and removing units from the set of neighbors. From the data collected during the experiments, it was found that the number of UAVs perceived during the flight is on average 3.87, which corresponds to 55% of the swarm. Analyzing the error on perception on each axis, it was found that the error on the *XY* plane is higher with respect to the z-axis. In fact, the error is on average 4.18 m on the x-axis, 5.35 m on the y-axis, and 1.49 m on the z-axis.

**FIGURE 9:**
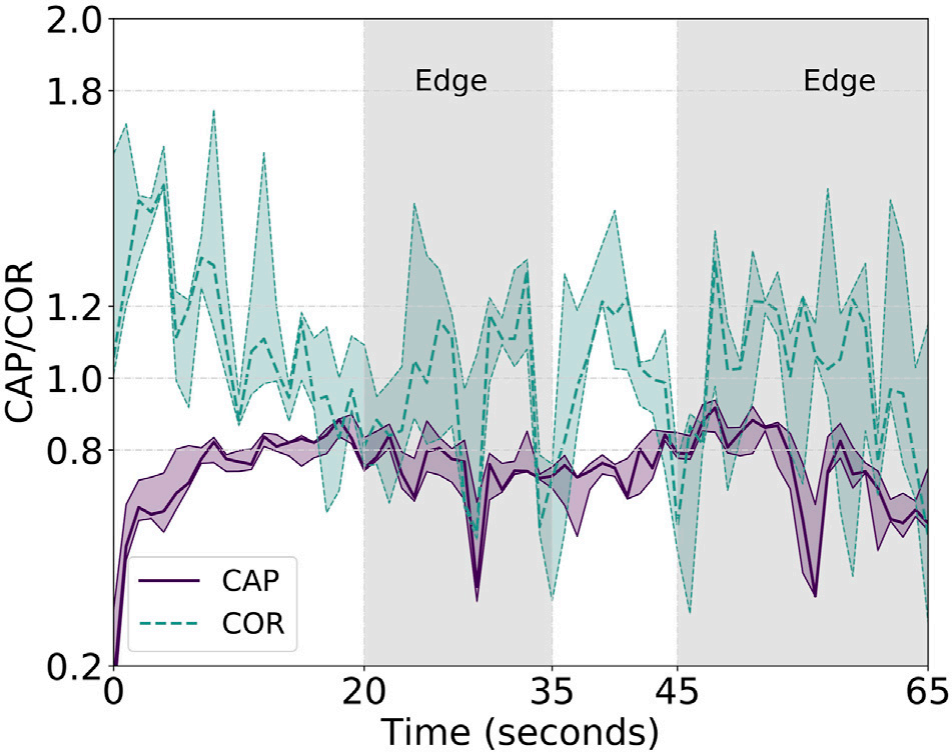
Reported CAP—solid purple line—metric and COR—dashed green line—metric for the real-field experiments with the UVDAR sensing technology. Higher y-values show better CAP performance. Close to 1 y-values show better COR performance.

CAP and COR analyses of these are shown in Figure 9. The cluster approach velocity of the swarm aligns with the one previously reported but with higher variance. In fact, we observe the same decrease in velocity at the edges during turns, followed by an immediate increase and group balancing effect induced by the flocking controller. The COR, however, shows a non-negligible imbalance in the unit velocities. Even in the presence of this, the swarm cohesiveness is not affected and, as shown in Table 1, the average intra-swarm distance and its variance over the three runs are comparable with the communication-full experiments.

### 4.5 Large swarm investigation

**FIGURE 10:**
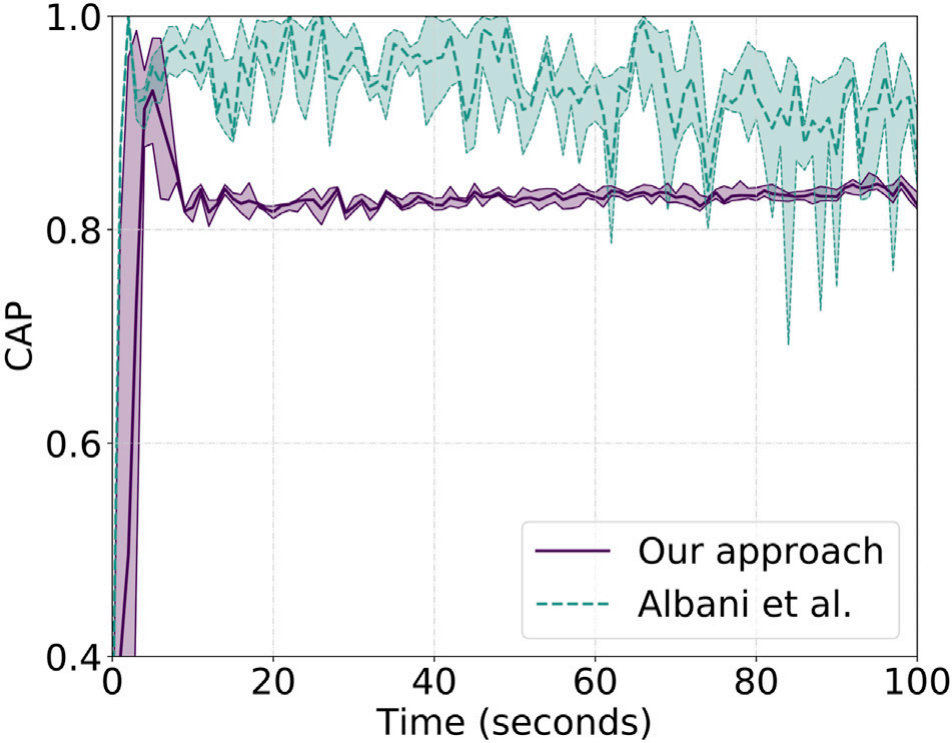
Reported CAP metric values for a single agent over 10 runs both for “our approach”—solid purple line—and the work of Albani et al. (2022)—dashed light-green line. Higher y-values show better CAP performance.

**FIGURE 11:**
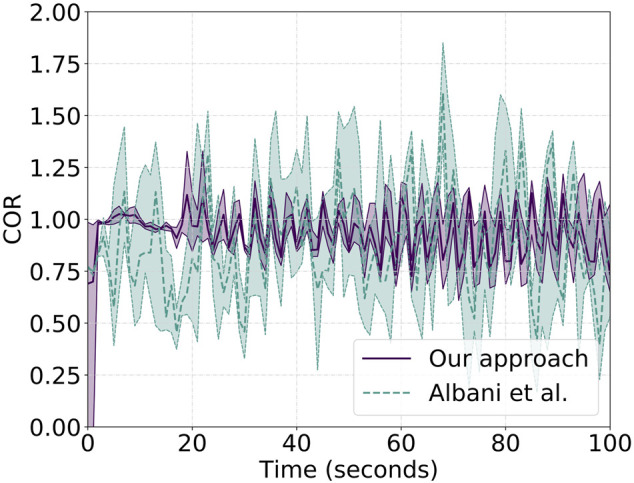
Reported COR metric values for a single agent over 10 runs both for “our approach”—solid purple line—and the work of Albani et al. (2022)—dashed light-green line. Close to 1 y-values show better COR performance.

In this section, we focus on the scalability of the proposed approach and evaluate the performances of our solution in larger swarms of 25 units fully connected by communication. For this evaluation, we rely on the same simulation environment previously used to replicate the field experiments and implement a second approach (Albani et al., 2022) for comparison with the newly proposed one. For each approach, we run a total of 10 experiments. At the beginning of each experiment, drones are spawned in a grid fashion and in predefined locations 10 m apart from each other, with a uniform random displacement of 3 m around the point. Next, the swarm is given a common way-point placed at a 1-km distance and a maximum velocity *V*_max_ of 8 m/s. The parameters for the approach proposed in this paper are identical to those used during the field experiments: the distance among the agents is 10 m and values for the Gaussian kernel are *σ*_1_ = 2, *σ*_2_ = 4, and *σ*_3_ = 6. On the other hand, for the setup of the competing solution, we follow the same setup proposed in the original work and set the distance among the robots to be 10 m. Results are shown in Figure 10 for the cluster approach velocity—CAP—while Figure 11 shows the correlated unit flocking velocity—COR. In both plots, a purple solid line identifies the performances of “our approach,” while a dashed light-green line is associated with the performance of the competing approach. We note that both plots are cut after 100 s and before reaching the final position. This is done on purpose due to the fact that over the 10 runs, the swarm does not show identical behavior and reaches the endpoint at different time instants, generating high noise and variance, providing no information about performances on the long run. In other words, we only focus on what can be thought as the initial steady state part of the behavior, leaving out the final phase.

Analysis of the CAP shows that our approach is characterized by a lower average velocity than the other approach that is, however, balanced by a non-negligible lower metric variance. This is related to the absence, as shown in Albani et al., of a proper balancing action as the one introduced by the importance weight. The work by Albani et al. presents a simple linear combination of the components that, on one side, increases the average CAP metric value but, on the other side, fails to smooth agent interactions. From a practical point of view, the latter means that the swarm center of mass moves at a roughly constant speed, while the competing approach is characterized by strong oscillations and sort of intermittent motion. This, caused by sudden acceleration and deceleration, generates a disruption in the swarm motion that might cause collisions and swarm separation. This is indeed shown in Table 2. The first row of the table presents results for “our approach,” and we note the complete absence of collisions and no separation—only one cluster is detected throughout the experiment. The bottom row, instead, reports the results for the comparing approach that, at a speed of 8 m/s, is not able to avoid separation and collisions. Indeed, over the 10 runs, the latter shows an average of 6.88 agent collisions with a group separation creating roughly two separate clusters every two runs. We also highlight how, from an energy efficiency point of view, a smoother cruise increases the time of flight. A property of the system is drastically linked to the change of thrust required for sudden acceleration and deceleration. Figure 11 confirms the CAP analysis and adds further strength to the aforementioned statements showing that single-drone and swarm velocities are not aligned toward the same direction. This is true for both approaches, but while “our approach” has a lower COR error of 0.15 on average, the other approach shows an error of 0.48.

**TABLE 2:** Reported average number of detected clusters and collisions over 10 runs for “our approach” and the work of Albani et al.

	Clusters	Collisions
Our approach	1	0.0
Albani et al. (2022)	1.6	6.88

## 5 Conclusion and future work

In this work, we presented a novel controller for a swarm of unmanned aerial vehicles. The proposed mechanism builds upon the virtual potential field theory and presents an automatic tuning mechanism of the forces that has been proven to work well both in simulation and in real-word settings. The experiments, performed with different maximum velocities and different group sizes, confirmed the replicability and effectiveness of the approach. As demonstrated in the paper, by only relying on local interactions and due to smooth tuning of these, the flocking controller also offers robustness and adaptability to robot faults, noisy and absent readings induced by blind spots in the perception. Furthermore, it has been shown how input abstraction allows for different sensors to be integrated with the approach. In fact, the only input required by the algorithm is a set of spatial coordinates with no information about the neighbors’ directions, environment boundaries, or anything similar. This aligns not only with common GPS readings but also with depth cameras, ultra-wide bands, and ultra-violet sensors.

Overall, we proposed a light-weight approach with potentially no upper-bound limit on the scalability. Along with the ease of implementation, we believe that these make our solution appealing to all those applications and platforms that require high computational power for other tasks and leave out a small percentage of it for group behaviors. Unmanned aerial systems fall within this category.

Among all the possible improvements and further analyses, two immediate future works we intend to proceed with are as follows: to test the proposed solution in the presence of obstacles and to improve the speed and stability of the controller. We aim at integrating external objects in the controller by acting directly on its structure, allowing for extra repulsive elements to be considered—similar to neighbor collision avoidance. On the other side, increasing the stability and the speed of the swarm will require a deeper analysis of the higher-order dynamics of the system. In particular, direct acceleration control of the units appears to be a possible solution to smooth the overall controller and deal with oscillations. Indeed, during real-world tests, we observed that the control input model—as introduced in Section 3—plays a major role in smoothing the oscillations. An aggressive low-level controller on a capable drone, due to it being more reactive, makes the overall approach more susceptible to noise and misreadings, thus leading to an increase of the oscillations. A less aggressive controller on a low-performing platform, on the other side, translates to smoother behavior—due to lack of performances—but also reduces the overall efficiency of the approach. To this end, we are working on an improved version of the proposed controller which will leverage third-order dynamics and generate faster, smoother, and more controllable behaviors.

Lastly, we observe that the use of different sensors yields different challenges among which we identified the presence of blind spots. Even though the latter was introduced by our hardware setup and can be mitigated by a better disposition of the cameras, it requires further analysis as we believe in the interest of the scientific community to study how the controller reacts to edge perceptions.

## Data Availability

The original contributions presented in the study are included in the article/Supplementary Material, further inquiries can be directed to the corresponding author.
